# Cure rate and associated factors for children 6–59 months with severe acute malnutrition under the out patient therapeutic care programme in the health centres of Kabale District in Southwestern Uganda: a cross sectional study

**DOI:** 10.1186/s40795-022-00560-5

**Published:** 2022-07-22

**Authors:** Vincent ssekajja, Henry Wamani, Freddy Eric Kitutu, Abel Atukwase

**Affiliations:** 1 Department of Community Health and Behavioural Sciences, School of Public Health Makerere University, Kampala, Uganda; 2 Department of Pharmacy, School of Medicine and Surgery Makerere University, Kampala, Uganda; 3 Department of Food Technology and Nutrition, School of Food Technology, Nutrition and Bio-Engineering Makerere University, Kampala, Uganda

**Keywords:** Malnutrition, Outpatient Therapeutic Care, Cure rate, Kabale district

## Abstract

**Background:**

Severe acute malnutrition (SAM) is one of the leading causes of morbidity and mortality among children below five years with sub-Saharan Africa being the most affected. In Kabale district, SAM affects 2.9% children under the age of five. Uganda government introduced Outpatient therapeutic care (OTC) programme in all health centre level III and IV of Kabale. However, there was limited information about the cure rate and its associated factors among children under the programme hence the cause for the study.

**Methods:**

A retrospective cross-sectional study was carried out on records of children 6–59 months (*n* = 637), presenting with SAM on OTC programme in the health centres of Kabale between 2013 and 2015. Data on cure rate (outcome) and other independent factors were collected, cleaned in excel and then exported into STATA 12 for analysis. Univariate, bivariate and logistic regression analysis was run to generate frequencies and factors associated.

**Results:**

The cure rate was 36.3% (*n* = 231 cases) with a median recovery time of 21 days. The default rate was 58.6% (*n* = 373 cases) while the non-response and death rate were 0.6% (*n* = 4) and 1.1% (*n* = 7) respectively. Source at admission (Adjusted Odds Ratio [AOR] = 0.1, 95% CI 0.0, 0.7, *p* = 0.012), Weight at admission (AOR = 0.5, 95% CI 0.0, 0.9, *p* = 0.014) and Number of visits to the program (AOR = 14.9, 95% CI 9.3, 24.2, *p* = 0.040) were positively associated with cure rate of SAM children on OTC programme in Kabale.

**Conclusion:**

Overall the cure and default rate for children on OTC programme in Kabale were significantly higher than national and international standards making the findings quite alarming. However, the weight of the child at admission, the number of visits to the programme to receive services and the source where the child was coming from were very important determinants of cure rate. To improve the cure rates of SAM children in Kabale, there is need for policy makers and programme implementers to think about a community based management of severe acute malnutrition program approach.

## Introduction

Severe Acute Malnutrition (SAM) is defined as very low weight-for-height (≤ -3SD) that is characterized by visible severe wasting or by the presence of nutrition Oedema and an upper arm circumference of less than 115 mm in children 6–59 months (WHO, WFP and UNICEF, [29]; Hobbs and Bush [4]). SAM is a global public health problem which affects an estimated 45.4 million children under five of which 12.1 million children are Africa (UNICEF, WHO and World Bank, [32]). Directly or indirectly, SAM is responsible for 53% of deaths of children under-five in developing countries (UNICEF, [27]; Collins, [24]) and the short term consequences, including compromised brain development, reduced growth and changes in body composition and metabolic programming among the survivors who may also end up growing up into poorly nourished adults (Shrimpton, [22], Mercedes et al., [14], Obaid, [19]).

According to a longitudinal study carried out by the World Health Organization and UNICEF in 2007, Uganda had a mortality rate of 12% among the under-fives due to SAM. The UDHS 2016 highlights the problem of malnutrition with stunting, wasting and underweight existing at a rate of 29%, 4% and 11%, respectively (UDHS, [26]). Current data shows that 3.5% of children under five in Uganda are still wasted (UNICEF, WHO and World Bank, [32]).

In 2010, OTC programme was introduced in all the health centres Level III and IV in Kabale, as it ensures timely detection and management of children with severe acute malnutrition (Ferguson et at., [20]). All children who had SAM with no medical complication, had appetite to eat the Ready-to-Use therapeutic feed (RUTF), those discharged from ITC irrespective of their anthropometry were admitted into the programme. Children under five having MAM with HIV or TB are also managed under OTC according to the national IMAM guidelines of 2016. In the program, children attend OTC centres bi-weekly to receive their RUTF (Plumpy nut) supplies and a course of routine medications including Vitamin A, amoxicillin, antimalarial drugs and measles vaccine to manage SAM without complications at home. The clients exited the program as either cured (WLZ/WHZ ≥ -2 z- score or MUAC ≥ 12.5 cm, dead, Non-response if failed to reach exit criteria after 3 months or defaulter if absent for two consecutive visits (IMAM guideline, [10]).

Nevertheless, in 2014, the GAM situation in Kabale district persisted at 3.8% and SAM at 2.9% despite the intervention (Wamani, [28]). Since the introduction of the programme, there has not been a comprehensive study conducted to evaluate its cure rate and associated factors and hence the cause for the study.

## Methodology

### Study design

A retrospective health centre based cross-sectional study design was used.

### Study site

Kabale district is approximately 420 km from Kampala the capital city of Uganda (Kabale district statstics report, [11]) and has an estimated population size of 248,700 people of whom 120,000 are males and 128,700 are females according to Uganda bureau of statistics 2020, Kabale. As a district, food insecurity, low socioeconomic staus, sub-optimal infant and young feeding practices, lack of quality drinking water and geographical challenges are some of the risk factors for malnutrition (Bakusuba, et al., [30] and Abaasa, et al., [31]).

This study was carried out in health centers Level III (*n* = 23) and IV (*n* = 07) which are running this therapeutic feeding programme in Kabale district located in Kigezi sub-region, south western Uganda.

### Selection of health centres that participated in the study

All the health centres were purposively selected because they are running the OTC programme. A total of 17 health centres were selected to participate in the study. Twelve (12) were health centre IIIs and 5 were health centre IVs.

### Source and population

All records of children 6–59 months treated under the programme between 2013 – 2015.

#### Inclusion and exclusion criteria

Records of children 6–59 months admitted to the OTC programme between 2013 to 2015 were included; children with incomplete records were excluded.

### Sample size (calculation/estimation)

The study sample size was calculated based on Kish and Leslie’s formula (Negash et al., 2015).

$$\mathrm N=\frac{\mathrm Z^2\mathrm P\left(1-\mathrm P\right)}{\mathrm d^2}=\frac{{1.96}^2\times0.85\left(1-0.85\right)}{{0.03}^2}=544\;\mathrm{Children}$$

Where N is sample size and d is the margin of error.

Z is cut off point at 5% level of significance.

P is prevalence of cure rate (85%) obtained from a similar program in Kabongo in 2011in the under five children with SAM (ACF, [1]).

By adding 15% for compensation of missing data (Tefera, et al., 2014), the final sample size was.

*N* = 625 children.

A roaster of eligible children was generated from the registration logbooks and only 637 children were found. The total number of eligible children generated being close to the calculated (625), all of them were enrolled into the study.

### Data collection procedure and quality control

A checklist was used to collect data from the OTC nutrition registration logbook for children aged 6–59 months who had SAM and were admitted to the program between July 2013 to January by a team of 5 trained nutrition research assistants. The quality of data was ensured by thorough training of research assistants who were nutritionists and also by checking all the questionnaires for completeness at every end of the day’s data collection.

### OTC outcome indicators evaluated in the study

Five outcome indicators were evaluated in this study: cure rate, death rate, default rate, non-response rate and referral rate (Table 1). In addition, the average length of stay (days) on the programme before discharge was also evaluated.

**Table 1:** Definition of OTC programme outcome indicators based on National IMAMGuidelines of 2016

Outcome Indicator	Definition
Cured	Attained weight for height/length (WH/L) Z score > -2 SD or Normal mid upper arm circumference (MUAC) (> 125 mm) cut off for two consecutive visits
Non response	Not reached target weight or MUAC without aggravating conditions for 3 months on the program
Defaulter	Patients missing two consecutive visits while on the program
Dead	Patient died while on the program
Transfer/Referral to ITC	Static (WH/L) z-score or MUAC or weight loss for two consecutive visits or not responding to treatment

Source: IMAM Guidelines for Uganda [10]

### Data management and analyses

Quantitative data was entered into Microsoft Excel, cleaned and then imported into STATA version 12 for analysis. univariete analysis was run to generate frequencies and percentages of the programme outcome indicators (cure rate, death rate, default rate, non-response rate and referral rate) together with other general characteristics of the study population. All the factors that were found to be associated with the cure rate of children in bivariate analysis were further analysed using multivariate analysis to assess the true factors associated with cure rate. Adjusted Odds Ratios (AOR) together with their respective 95% confidence intervals were reported in the table. *P*-Values < 0.05 were considered to be statistically significant.

## Results

The socio-demographic characteristics of the children (6–59 months) were summarized in Table 2. The results indicate that out of the total 637 children, 54% (*n* = 343) were females, 47.4% (*n* = 302) children were aged 6–12 months. Most of the children 54% (*n* = 344) were being managed from health centre level IV and the average weight for the children at admission was 7.1 + 2.1 kg with over 64% (413) having an admission weight of 6 – 10 kg. The average height for the children was 71.5 + 27.8 cm with the majority (45.2% (*n* = 288) children in the range of 60–69.9 cm.

**Table 2:** Showing the socio-demograpghic characteristics of children enrolled on OTC programme

Characteristic	**Percentage (Frequency) *N* = 637**	**Cut-offs**
**Sex**		
female	54 (343)	
male	46 (292)	
**Age in months**		
6–12	47.4 (302)	
13–18	24.8 (158)	
19–24	13.3 (85)	
25–59	14.4) (92)	
**Height in cm**		
50–59.9	6.1 (39)	
60–69.9	45.2 (288)	
70–79.9	39.7 (253)	
80–150	8.9 (57)	
**Weight at admission**		
3–5.9	26.5 (169)	
6–9.9	64.8 (413)	
10–14.9	7.7 (49)	
15–22	0.9 (6)	
**Nutrition status**		
SAM without oedema	71 (452)	< -3 SD
SAM with oedema	3.9 (25)	< -3 SD
MAM with HIV	2.5 (16)	(≥ -3 SD & < -2 SD
MAM with TB	22.6 (144)	(≥ -3 SD & < -2 SD
**HIV status**		
Positive	4.4 (28)	
Negative	68.3 (435)	
Unknown	27.3 (174)	
**Source of admission**		
Community	96.7 (616)	
Health centre	3.3 (21)	
**Readmission**		
Yes	8.5 (54)	
No	91.5 (583)	

The study findings indicate that only 4.4% (*n* = 28) were HIV positive. About 2.5% (*n* = 16) of the HIV positive children were moderately malnourished. About 2% (*n* = 174) of the children who participated in the study did not have HIV test results in their records. The majority of the children 71% (*n* = 452) had SAM without Oedema. Admission was more from the community 96.7% (*n* = 616) and only 8.5% (*n* = 54) of the children were re-admitted into the program.

### Programme outcome indicators in comparison with national IMAM and sphere standards

The results of the programme indicators presented in Table 3 show that the cure rate was at 36.3% (*n* = 231) while the death rate was at 1.1% (*n* = 7). The results indicate that there was a high default rate of 58.6% (*n* = 373). The non-response rate was at 0.6% (*n* = 4). The results also indicate that the average length of stay on the programme was 21 days with about 20% (*n* = 124) staying on the programme for more than 30 days and referral rate at 3.5% (*n* = 22). The average number of visits made by the children under the programme was 1.4 + 1.7 visits.

**Table 3:** Programme outcome indicators in comparison with Uganda national IMAM guideline and international sphere standards (Sphere project., [23])

Indicator	Results *N* = 637 Percentage (Frequency)	IMAM Standard	Sphere Standard	
			**Acceptable**	**Alarming**
Cured	36.3% (231)	˃75%	> 75%	< 50%
Died	1.1% (7)	< 5%	< 10%	> 15%
Defaulted	58.6% (373)	< 15%	< 15%	> 25%
Non-respondent	0.6% (4)	< 10%	15%	
Referred	3.5% (22)	< 10%		
Average Length of stay	21 days	< 60 days	< 8 weeks	> 8 weeks
Average Recommended number of visits	1.4	4 visits		

### Results of bivariate and logistic regression analysis to establish the factors associated with cure rate are summarized in Table 4.

**Table 4:** Results of bivariate and multivariate analysis indicating factors associated with the Cure of children from SAM in the Health centres of Kabale district between 2013 and 2015 (*N* = 637)

Variable	Unadjusted Odds Ratios (95% CI)	*P*-value	Adjusted Odds Ratios (95% CI)	*P*-value
**Health centre**				
IV	1.0			
III	**0.6(0.4 – 0.8)**	**0.02**		
**Source of admission**				
Health centre	1.0			
Community	**0.3(0.8 – 0.9)**	**0.033**	**0.1 (0.0 – 0.7)**	**0.012** ^**a**^
**Weight at admission**				
3 – 5.9	1.0			
**6 – 9.9**	**0.5(0.4 –0.7)**	**0.0006**	**0.5 (0.0 – 0.9)**	**0.014** ^**a**^
10 – 14.9	2.5(0.3 – 1.2)	0.118		
15 – 22	2.2(0.0 – 1.9)	0.139		
**No. of Visits**				
0	1.0			
**1 – 4**	**6.9(4.0–11.9)**	**0.001**	**14.9(9.3 – 24.2)**	**0.040** ^**a**^
**5 – 8**	**5.8(2.3–14.9)**	**0.001**		
**Length of stay (days)**				
0	1.0			
**15– 30**	**7.0(4.1–12.3)**	**0.0001**		
**31 – 120**	**5.3 (0.2—8.7)**	**0.0001**		
**Age**				
6 – 12	1.0			
13 – 18	0.8(0.6 – 1.2)	0.330		
**19 – 24**	**0.5(0.3 – 0.9)**	**0.010**		
25 – 59	0.8(0.5 – 1.4)	0.470		
**Sex**				
Female	1.0			
Male	0.8(0.6 – 1.2)	0.330		
**Height at admission**				
50 – 59.9	1.0			
60 – 69.9	0.6(0.3 – 1.3)	0.190		
70 – 79.9	0.5(0.3 – 1.0)	0.050		
80 – 150	0.6(0.3 – 1.4)	0.250		
**Readmission**				
Yes	1.0			
No	0.9(0.5 – 1.6)	0.680		
**HIV status**				
Positive	1.0			
Negative	0.9(0.5 – 2.1)	0.920		
Unknown	0.7(0.3 – 1.6)	0.860		
**Nutrition status at admin**				
SAM without oedema	1.0			
SAM with oedema	0.9(0.4 – 2.0)	0.730		
MAM with HIV	1.8(0.7 – 4.9)	0.230		
MAM with TB	1.1(0.8 – 1.7)	0.540		

^a^ Significant at α = 0.05

The bivariate analysis indicated that the health center where SAM management was done (OR = 0.6, 95% CI: 0.4—0.8, *p* = 0.02), the source where the child was coming from (OR = 0.3, 95% CI: 0.8—0.9, *p* = 0.033), the weight at admission (OR = 0.5, 95% CI: 0.4 – 0.7, *p* = 0.0006), the number of visits to the program to receive the services (OR = 6.9, 95% CI: 4.0 – 11.9, *p* = 0.001) and the Length of stay on the program (OR = 7.0, 95% CI: 4.1 – 12.3, *p* = 0.001) were associated with the Cure rate. After adjusting for any possible confounders in multivariate analysis, only the Source of admission (AOR = 0.1, 95% CI: 0.0, 0.7, *P* = 0.012), Weight at admission (AOR = 0.5, 95% CI: 0.0, 0.9, *P* = 0.014) and Number of visits to the program to receive the services (AOR = 14.9, 95% CI: 9.3, 24.2, *P* = 0.040) were the major factors influencing the recovery of children from severe acute malnutrition under the OTC programme.

## Discussion of results

The findings showed a cure rate of 36.3% very low compared to both the national (IMAM) and international (Sphere) standards that recommend a Cure rate of greater than 75% hence in an alarming state. The findings further indicated a lower Cure rate than in similar studies done in Ethiopia, Pakistani and Zambia (Teshom  et al., [9], Eleanor et al., [7],  Atnafe et al., [5] and Mwanza et al., [15]). This relates to findings by Zebenay and colleagues who found a low recovery rates in a similar programme in Ethiopia and suggested non adherence to treatment guidelines by the caretakers, sharing food at home, high burden of comorbidities as well as inappropriate feeding with RUTF as the likely causes. However, the results of this study showed a slightly higher Cure rate than the one observed in a similar study done in Ghana in 2015 with a Cure rate of 33.6% (Mahama et al., [13]). The reason for this could be due to the number of factors like differences in the socioecomic status, geographical challenges as well as variation in the clinical expertise of the health care providers as observed by Abaasa and colleagues (Abaasa, et al., [31]).

The Death rate, the Non-response rate and the average Length of stay were within acceptable levels based on both the national and international sphere standards. Death rate in the study were in line with studies done in Ethiopia (Shanka et al., [18]; Atnafe et al., [5]; Mulugeta et al., [16]) and in Pakistan (Eleanor et al., [7]). It was also noted that the Death rate in the current study was much lower than the one observed in Malawi (Saddler, [12]). These findings could be associated with proper adherence to the treatment protocol under the OTC programme.

The overall Default rate in this study was way out of the national and the international standards and also higher than findings in similar studies in other countries like Ghana (Mahama et al., [13]), Ethiopia (Chane et al., [25]; Mulugeta et al., [16]), and Pakistani (Eleanor et al., [7]). However, the Default rate was found to be close to a recent study done in Ghana by Mahama et al. in [13] and the one done by Action against Hunger (2011) in Moroto Karamoja sub-region in North Eastern Uganda. In general, the default rate was very unacceptable and this could be due to long distances walked by the caretakers to reach the health facilities as indicated by ACF in Karamoja sub-region Northern Uganda.

The average Length of stay on the program for children who cured from SAM was 21 days (3 weeks) which was within the acceptable national and international standards and this agrees with recent studies done in Ghana (Mahama et al., [13])] and in Ethiopia (Chane et al., [25]; Kabeta and Bekele, [2]; Muluken B.M, [17]). This was related to the caretaker’s compliance to the OTC programme treatment protocol.

The Nonresponse to treatment was within the acceptable levels and this could be related to early health seeking behaviours by the caretakers which reduces long stay to the OTC programme which is in line with sphere standards (2004) where it’s noted that seeking treatment late is associated with delayed or long stay on the programme.

### The factors predicting cure of children from severe acute malnutrition under the OTC programme

The source where the child was admitted from was significantly associated with the cure of the child from severe acute malnutrition. It was seen that children who were admitted from the community were 0.3 times less likely to cure from SAM than those admitted from other health facilities and finding was similar to studies done in Zambia and Ghana (Michelo and Muyode, [6]); Hamulembe, [21]) and this was because of the poor health seeking habits by the caretakers who bring children to the health facilities when it’s already late. However this study was contrasting with a similar study done in Ghana by Mahama et al. [13] which did not find any association between source of admission and cure of children from severe acute malnutrition.

The weight of the child at admission was also associated with the child’s Cure from SAM. The admission weight between 6—10 kgs were 0.5 times less likely to cure from SAM as compared to those between 3–6 kgs with reasons related to breastfeeding. When cross tabulations were run, it was noted that majority children between the ages of 6 – 12 months were falling in the weight ranges of 3 – 6 kg meaning that breast feeding children if complemented with RUTF can have better cure rates than elder children. However this was contradicting with findings of the study done in Ghana by Mahama et al. in [13].

The number of visits the child made to the programme to receive supplies was positively associated with the cure of children from severe acute malnutrition. Children who made between 1 – 4 visits where 4 visits are the standard recommended were 6.9 times more likely to cure from SAM than those who did not make a single visit and this was in line with a study done in Ghana and Ethiopia (Mahama et al., [13] and Hamulembe, [21]). The reason was compliance to treatment that enabled cure of children.

Other factors like Length of stay on the programme, age of the child at admission and the health centre where the child sought treatment seemed to be associated with cure of children from severe acute malnutrition in Bivariate analysis but it was because of confounding that they appeared so. However these factors have been found significant in other studies done in south Sudan, Malawi and Zambia by other countries but in this particular one they were not predicting cure rate of children (Taylor, [3]; Saddler, [12]; Michelo and Muyode, [6]). This could be due to the differences in geography of the area, clinical expertise of programme managers as well as adherence to the ready to eat therapeutic feeds.

### Study limitations

The absence of information on weight gain, history on breast feeding, distance from home to the health centre, maternal education, household food security and wealth index limited the understanding of factors affecting recovery of children from SAM.

## Conclusion

From this study, it can be concluded that the OTC programme in Kabale district was not performing well looking at the programme outcomes especially cure rates and default rates. There is need for policy makers and programme implementers in the district to think about a community based management of SAM programme where children will be identified and treated early as well as reduction in the walking distances to seek treatment which are associated with positive outcomes. There is also need for capacity building among the health workers to improve delivery of OTC services in the health centres as well as increase programme support and supervision.

## Data Availability

Data will be made available upon request from the corresponding author.

## Abbreviations

OTC: Outpatient Therapeutic Care
IMAM: Integrated Management of Acute Malnutrition
AOR: Adjusted Odds Ratios
SAM: Severe Acute Malnutrition
RUTF: Ready-to- Use Therapeutic Food
CI: Confidence Interval
ITC: Inpatient Therapeutic Care
ACF: Action Against Hunger
OR: Odds Ratio
MAM: Moderate Acute Malnutrition
HIV: Human Immune-deficiency Virus
MDG: Millennium Development Goals
HC: Health Centre, SD: Standard Deviation
MUAC: Mid-Upper Arm Circumference
BMI: Body Mass Index
WFH: Weight For Height
FANTA: Food and Nutrition Technical Assistance
UNICEF: United Nations Children Education Fund
